# Is Gestational Diabetes Mellitus an Important Contributor to Metabolic Disorders in Trinidad and Tobago?

**DOI:** 10.1155/2009/289329

**Published:** 2009-05-11

**Authors:** M. Clapperton, J. Jarvis, K. Mungrue

**Affiliations:** Faculty of Medical Sciences, The University of the West Indies, EWMSC, Mt. Hope, Trinidad and Tobago

## Abstract

*Objective*. To investigate the incidence of Gestational Diabetes Mellitus at the Mt. Hope Women's Hospital and to describe its epidemiological pattern. *Design*. A retrospective observational study (Jan 2005 to Dec 2007). *Setting*. A teaching hospital of The University of the West Indies. *Population/Sample*. Pregnant women who gave birth. *Methods*. A sample size of 720. The variables analyzed were: age, ethnicity, BMI of mother, family history of diabetes; history of GDM, obstetric history, birth weight and APGAR score of infant. *Main Outcome Measures*. (1) Incidence of cases of GDM. (2) Impact of the measured variable. Chi-squares, odds ratios and logistic regression were performed. *Results*. The incidence of GDM was 4.31% (95% C.I. 2.31%, 6.31%). The proportion of GDM patients for the years 2005, 2006, and 2007 were 1.67%, 4.58%, and 6.67%, respectively. Age, Obesity Ethnicity, Family history of diabetes and a history of GDM were determined risk factors. Associations between GDM and (1) Mode of Delivery and (2) APGAR score of the baby were found. *Discussion & Conclusion*. There was an apparent increase in the incidence of GDM. Additional studies should be conducted to measure the occurrence of GDM in Trinidad and Tobago. Efforts to promote public awareness and a healthy lifestyle should be made to reverse this trend.

## 1. Introduction

Pregnancy is a diabetogenic state whereby insulin sensitivity decreases with advancing gestational age [1]. Gestational diabetes mellitus (GDM) is defined by the World Health Organization (WHO) as carbohydrate intolerance resulting in hyperglycaemia of variable severity with onset or first recognition during pregnancy [2, 3]. This may be due to previously unrecognized pregestational DM (pre-GDM) or true GDM, that is, undetected DM type 2 occurring prior to and independent of the pregnancy. The WHO, further defines GDM as a fasting plasma glucose level ≥7.0 mmol/L (≥1.26 g/L) or a casual plasma glucose ≥11.1 mmol/L (≥2.00 g/L), confirmed on a subsequent day [3].

The risks to the foetus include increased birth weight and risk of perinatal morbidity and mortality [3]. Furthermore, GDM is associated with increased risk of maternal morbidity and the development of type 2 diabetes mellitus (T2DM) [4, 5]. Maternal age, a first degree relative with diabetes, ethnicity, and obesity are primary risk factors for developing GDM. 

In 2001, GDM affected up to 14% of the pregnant population in the United States [6]. Also, the risk of GDM recurring has been reported to be 60% to 90% [6]. As much as 50% of women with GDM will develop overt T2DM after 25 years [7]. In 2000, approximately 19 million people from Latin America and the Caribbean suffered from T2DM [6]. This number is expected to double by the year 2025 [8].

A literature review revealed no published data on the prevalence of GDM in Trinidad and Tobago. Hence, it is important to measure the occurrence of GDM in order to (1) provide evidence on the magnitude of the problem and its impact on the delivery of care, particularly enhanced screening and intervention at the primary care level and (2) guide policy and decision making especially when allocating precious resources. The purpose of this study is to estimate the prevalence of GDM in women who have given birth at the Mt. Hope Women's Hospital and describe its epidemiological pattern.

## 2. Methodology

This study utilized retrospective clinical data obtained from patients admitted and followed through to delivery at the MHWH, a teaching hospital, which services clients mainly from the Northern half of the island for the period January 2005 to December 2007. Previous studies reporting any prevalence of GDM in the studied population were not available. Therefore, in order to validate our findings, a census was conducted as a pilot study to determine the incidence of GDM cases occurring between January and May 2008. The admission registers on the antenatal wards were used to identify both confirmed and queried diagnoses of GDM cases. Each file was reviewed to confirm the presence of a diagnosis of GDM and followup notes till delivery. 

Only individuals with known risk factors were usually subjected to a 50 g oral glucose challenge, if abnormal a 75 g oral glucose tolerance test (OGTT) is done to confirm the diagnosis. All other patients only have a random blood glucose test done at their first visit to the institution. If random blood glucose tests for nonrisk patients are normal, another random blood glucose test is conducted at 36 weeks gestation. Pregnant women who meet WHO criteria for diabetes mellitus or impaired glucose tolerance (IGT) are classified as having GDM. After the pregnancy, the woman is reclassified as having either DM, or IGT, or normal glucose tolerance based on the results of a 75 g OGTT six weeks or more after delivery [2]. The diagnosis of GDM for this study was based on the diagnosis made and recorded by attending physicians which were then extracted from the patient's file. 

The Monthly Report of Inpatient Services was obtained from the records department to determine the total number of deliveries for the period which was then used as the denominator for calculating the rate of occurrence of GDM for the period January to May 2008. From the 1896 patients admitted to the hospital during January to May 2008, the proportion of GDM patients was 2.9% *≈* 3%. This rate was used as the estimated occurrence of GDM in other years. The birth registries provided the total number of deliveries for each year. The total number of deliveries for the 3 years (January 2005 to December 2007) was 12 655 from which a minimum sample size of 715 was calculated using the formula:

(1) $$n\;\geq \frac{4Z_{\alpha /2}^{2}p\left(1-p\right)}{w^{2}}$$

(estimated prevalence *p* = 3%, *α* = 0.05, and precision *w* = 2.5%) [9].

Each file was selected using systematic sampling [9]. In our setting, there are no digitally stored patient records. Only hardcopies are filed and stored in the records department. These files were not listed in any particular order in the registry. All deliveries for each year were recorded in a delivery book in an arbitrary sequence. On average, 20 files were able to be retrieved per day, thus restricting the possibility of evaluating the entire population (12 655). Since the total number of deliveries for each year varied by less than 2%, the subset taken for each year was uniform. The first file number was randomly selected. Every subsequent *n*th file was selected until 238 files were selected for each year. However, 240 files were selected to increase the sample size to 720. “*n*” was calculated by dividing the population of births for the three years by our sample size estimate (12655 † 714 ≈ 20), thus every 20th file was selected. Each participant selected was reviewed to determine eligibility for entry into the study. 

The exclusion criteria were (1) women who gave birth at the MHWH before January 2005 and after December 2007, (2) miscarriages before 28 weeks of gestation, and (3) women with a previous diagnosis of diabetes (Type 1 or Type 2). If a record satisfied any of the exclusion criteria, it was discarded and replaced using the same procedure previously outlined, by retrieving the subsequent 20th file, that is, after the last selected file for the respective year. Similarly, if a patient's record was incomplete such that age, gestational age at delivery, date and mode of delivery, parity and gravidity were omitted, it was also replaced. Because the likelihood of developing GDM in subsequent pregnancies for women with a history of GDM was also being assessed, each participant was treated as a new and independent case regardless of a previous inclusion into the study in a previous year. 

To control and validate the data extracted, an initial trial run was performed by the authors of the study to determine the feasibility and reliability for abstracting the data. Following that, abstractors were trained and monitored to prevent disparities in data collection. Two persons were assigned to inspect each file retrieved and recheck data upon entry. Also, the authors were always present to oversee data collection. All data was retrieved, stored, and analyzed using the Statistical Package for Social Sciences (SPSS) Version 15.0. 

The variables analyzed were age, ethnicity, gravidity, parity, BMI of mother at booking or ≥34 weeks gestation, family history of diabetes; history of gestational diabetes, gestational age at delivery, mode of delivery (Caesarian section/Vaginal delivery), complications at delivery, birth weight of infant, and APGAR score of infant. A binary logistic regression analysis was performed to determine the extent of association the investigated risk factors had with GDM. Potential confounders leading to elevated blood glucose levels were (1) pathological conditions, for example, acromegaly, and hyperthyroidism and (2) medical/surgical treatments, for example, pancreatectomy, glucocorticoid treatment, and *β*-adrenergic agonists. The outcome variable was the proportion of participants who met the WHO criteria for GDM among mothers who gave birth within the third trimester. The study was approved by the ethics committee of the University of the West Indies. 

## 3. Results

Of the 720 women who were eligible for entry into the study, 31 participants met the criteria for GDM. The overall proportion of women with GDM was 4.31% (95% CI 2.31%–6.31%) for the period January 2005–December 2007 (see Table 1).The proportion of GDM patients for the years 2005, 2006, and 2007 were 1.67%, 4.58%, and 6.67%, respectively, indicating an increasing trend. Using this trend, a predicted value of 9.31% was calculated for 2008. However, the actual value is expected to lie between 6.67% and 9.31%. 

The mean age of mothers in the sample was 30.4 years (95% C.I. 27.7, 33.1). The age range 35–39 had the highest proportion of GDM (Pearson Chi-Square *P* = .001). Additionally, it was 4 times more likely for GDM (OR 4.07 95% C.I. 1.96, 8.45) to occur in women aged 30–34 than women <30. However, women ≥35 were 8 times more likely to acquire GDM (OR 8.00 95% C.I. 2.17–29.5).

The sample population consisted of South East Asians (35.1%), Africans (38.5%), Mixed (19.2%), and other ethnicities (7.2%). South East Asians (*P* = .016) were 2.7 times more likely to develop GDM than Africans (OR 2.73 95% C.I. 1.17, 6.35). Also, South East Asians (*P* = .011) were 5.5 times more likely to develop GDM than Mixed (OR 5.521 95% C.I. 1.27, 24.07). 

A mean Body Mass Index (BMI) of 28.69 (95% C.I. 27.90, 29.48) was calculated for the 260 women who had consistently recorded measurements. Sixty-eight percent of these women were either overweight or obese. The association (*P* = .013) between women with healthy BMI's (18.4–24.9) and obese women (BMI > 30) revealed that obese women were 9 times more likely to acquire GDM (OR 8.97 95% C.I. 1.14, 70.58). Due to the large confidence interval, it is recommended that a larger study be done to strengthen this association. However, previous studies have also reported on this association. It was also observed that the proportion of GDM cases increased with increasing BMI (see Table 2).

Of the 720 women, 17.4% delivered via Caesarian Section (CS). Among the 31 diagnosed cases of GDM, 41.9% delivered via CS. It was 3.7 times more likely for those with GDM (*P* = .000) to have a CS (OR 3.72 95% C.I. 1.77, 7.81). The mean 5 minute APGAR score for the entire sample was 8.87 (95% C.I. 8.81, 8.92). Even though many studies have stated a normal APGAR score to be ranged 7 to 10, no association was found between the presence of GDM and a score below 7; due to the insufficient data obtained to analyze APGAR scores less than 7, a 5 minute APGAR less than 8 was subsequently utilized. Women with GDM (*P* = .023) were 4 times more likely to deliver a child with a 5 minute APGAR score of <8 (OR 3.98 95% C.I.1.77, 7.81) than women without GDM. The mean weight of the neonates at delivery was 3.05 kg (95% C.I. 3.01, 3.10). The mean weight of the neonates within those diagnosed with GDM was 3.12 (95% C.I. 2.84, 3.40). No significant association could be found.

Approximately 19.4% of women with GDM in their current pregnancy had a history of GDM. An association (*P* = .000) between history of gestational diabetes and currently developing GDM revealed that a woman with a history of GDM was 41 times more likely to develop GDM in her subsequent pregnancy (OR 41.10 95% C.I. 10.91, 154.87). Approximately, 34% of the entire sample had a family history of diabetes. This proportion increased when observing those with GDM (61.3%). A strong association between family history of diabetes and GDM (*P* = .001) showed that patients with a family history were 3 times more likely to develop GDM (OR 3.24 95% C.I. 1.55, 6.8). The results of binary logistic regression analysis showed associations between GDM and age, BMI, ethnicity, family history of GDM and history of GDM, and birth weight of infant (see Table 3).

## 4. Discussion

The proportion of women with GDM who gave birth at the MHWH during the period January 2005 to December 2007 was 4.3% (95% CI 2.31%, 6.31%). Regionally, Barceló and Rajpathak in 2001 reported a prevalence 4% among pregnant women in the Americas [10]. The prevalence of GDM may range from 1% to 14%, depending on the population studied and the diagnostic tests employed [10]. The proportion of birth mothers with GDM at the MHWH increased from 1.67% in 2005 to 6.67% in 2007 and is projected to reach 9.31% in 2008, that is, a 5.6-fold increase from 2005. Several factors may have contributed to this finding particularly (1) an increase in the awareness and diagnosis by physicians, (2) the establishment of a diabetic clinic in 2005, and (3) an actual increase in the incidence of GDM in the population. Since there was no information to suggest an increase in surveillance and any formal government interventions (e.g., with respect to screening) introduced to contribute toward a measured increase, such trend may will be due to a true increase in the incidence of GDM in the population. However, larger studies must be conducted to actually determine the reason for the trend observed.

Obesity in the Caribbean has doubled in ten years and is now common. The epidemic has been aided and abetted by the fast food industry, with a proliferation of fast food restaurants throughout the Caribbean. The transition of agriculturally based societies to service-based ones has taken its toll. With development, there has come increasing mechanization, a decline in manual labor, and improvements in transportation. Thirty-four percent of adults in Trinidad and Tobago are sedentary. Leisure-time physical activity in the region is the lowest in Trinidad and Tobago [11]. In keeping with this trend, it was observed that a large proportion of the study population had unhealthy BMIs, that is, 68% were overweight (BMI > 25) and 41% were obese (BMI > 30). 

Both obesity and a family history of T2DM represent important risk factors for the development of GDM [12]. It was found that obese women in this study were at the greatest risk of developing GDM (OR 8.97, *P* ≤ .05) compared to nonobese participants. Additionally, a 2006 study conducted in the United Kingdom demonstrated that insulin resistance was the most significant change which predisposed obese pregnant women to GDM [13]. Thus, we recommend that all patients who have their BMI measured to establish obesity and following delivery should be monitored and managed to achieve a target BMI of 18.5–24.9 kg m^−2^. Weight loss during pregnancy is not advisable as there are no evidence-based guidelines to support this practice. 

South East Asians (OR 2.73, *P* ≤ .05) were found to have a higher risk of GDM than Africans. Also, women with a family history of GDM (OR 3.0, *P* ≤ .05) or a past medical history of GDM (OR 41, *P* ≤ .05) were more likely to develop GDM than those without. It may be inferred that this was probably due to dietary differences and/or a genetic predisposition (familial T2DM). 

In accordance with past studies, women with GDM were more likely (OR 3.72, *P* ≤ .05) to have a caesarian delivery as opposed to a vaginal delivery. Other than macrosomia, the increased risk of caesarean delivery for women with GDM may also be due to clinical practices at the health institution and the rate of physician referrals to high-risk care [14]. When compared to babies born to non-GDM mothers, those born to mothers with GDM were at higher risk (OR 3.98 *P* ≤ .05) of having a lower five-minute APGAR score (<8). A low APGAR score usually implies infant mortality, neonatal neurologic morbidity, and poor long-term outcome. A 2001 study conducted in Sweden by Thorngren and Herbst reported that infants with a 5-minute APGAR score <7 were more likely to have seizures, intracranial haemorrhages [15].

Women 30–34 were found to be at a greater risk (OR 4.07 *P* ≤ .05) than those 15–29, and this doubled for women over 35 (OR 8 *P* ≤ .05). While older women appear more likely to develop GDM, this could be explained by multigravidity in older women as well as weight retention from previous pregnancies contributing to subsequent obesity.

 In conclusion, 4.3% of the women who gave birth at the MHWH during January 2005–December 2007 had GDM. Since the prevalence of GDM seems to be increasing at an alarming rate (1.6% in 2005 to 6.7% in 2007), it is crucial to heighten postpartum vigilance for T2DM via the commencement of early and long-term screening. Since other factors may have contributed toward this increase, it is thus recommended that larger studies are conducted in order to assess the magnitude of the problem and determine whether or not the trend observed is in fact due to an actual increase in the true occurrence of GDM. It is also proposed that the following WHO and American Diabetes Association recommendation be implemented: initiation of screening for T2DM at 6–12 weeks postpartum by either the measurement of fasting plasma glucose levels or the 75 g oral glucose tolerance test (OGTT) [16, 17]. If results are normal, then the patient should be reevaluated every 3 years; if impaired glucose tolerance is detected, annual screening is then recommended. GDM identifies a population of women at high risk for developing subsequent T2DM; it represents an early stage in the natural history of the disease and therefore provides an opportunity for early intervention and reduction of the national prevalence of T2DM [18, 19].

## Figures and Tables

**Table 1:** Demographics of study sample.

Number of cases (number with gestational diabetes)									Total
Age ranges									
	≤14	15–19	20–24	25–29	30–34	35–39	40+		Total

No. of patients	1	115 (3)	224 (6)	173 (5)	139 (5)	62 (9)	6 (3)		720 (31)

Gravidity ranges									

	Primigravida		Secundigravida		Multigravida		Grand multigravida		
	(0 previous		(1 previous		(2–4 previous		(5 or more previous		Total
	pregnancies)		pregnancy)		pregnancies)		pregnancies)		

No. of patients	210 (11)		186 (5)		209 (10)		115 (5)		720 (31)

Ethnicity									

	Indo-trinidadian	Afro-trinidadian	Caucasian	Chinese	Mixed	Unknown			Total

No. of patients	253 (19)	277 (8)	0	1	138 (2)	51 (2)			720 (31)

Weight of mother at booking									

	40–50 Kg	51–60 Kg	61–70 Kg	71–80 Kg	81–100 Kg	101–120 Kg	>121 Kg	Unknown	Total

No. of patients	17	71	111 (1)	109 (10)	132 (13)	35 (3)	9	236 (4)	720 (31)

Body mass index of mother at booking									

	<18.5	18.5–24.9	25–29.9	>30	Unknown				Total

No. of patients	9	72 (1)	72 (10)	107 (12)	460 (8)				720 (31)

Mode of delivery									

	Spontaneous		Caesarian						Total
	vaginal		section						

No. of patients	595 (18)		125 (13)						720 (31)

Weight of baby at delivery									

	<2.5	2.5 – 4.0	>4.0						Total

No. of patients	98 (7)	595 (21)	27 (3)						720 (31)

**Table 2:** Odds-ratios and Pearson chi-square tests.

			95% Confidence interval					95% Confidence interval	
	Pearson	Odds	Lower	Upper		Pearson	Odds	Lower	Upper
	chi-square	ratio				chi-square	ratio		

Age					Caesarian section				
30–34/<30	0.0001	4.07	1.96	8.45	*GDM/No GDM *	0.0001	3.72	1.77	7.81
>35/<30	0.0001	8	2.17	29.5	APGAR				
Ethnicity					< *7/ (7–10) *	—	—	—	—
*South-East Asian/Africans*	0.016	2.73	1.17	6.35	< *8/ (8–10) *	0.023	3.98	1.77	7.81
*South-East Asian/Mixed *	0.011	5.52	1.27	24.07	Hx of GDM	0.000	41.1	10.91	154.87
BMI					Family Hx of GDM	0.001	3.24	1.55	6.8
*Obese/Healthy *	0.013	8.97	1.14	70.58	Birth weight	—	—	—	—

**Table 3:** Binary logistic regression.

		B	S.E.	Wald	df	Sig.	Exp(B)	95.0% C.I. for EXP(B)	
								Lower	Upper
Step1(a)	Ages (15–30) & (>30)	−1.289	.446	8.358	1	.004	.276	.115	.660
	Healthy (18.4–24.9) &Obese (>30) BMI	.212	.069	9.385	1	.002	1.236	1.079	1.415
	Africans, South East Asians & Mixed	.683	.353	3.750	1	.043	1.981	1.092	3.956
	History of GDM	3.181	.809	15.473	1	.000	24.075	4.934	117.478
	Family history of GDM	.860	.451	3.627	1	.037	2.362	1.975	5.722
	Gestational age at delivery	.117	.102	1.301	1	.254	1.124	.920	1.373
	APGAR (<7) & (7–10)	1.298	1.722	.568	1	.451	3.662	.125	107.074
	APGAR (<8) & (8–10)	.883	1.288	.471	1	.493	2.419	.194	30.184
	Infant underweight, normal, overweight	−1.279	.596	4.608	1	.032	.278	.087	.895
	Constant	−5.500	3.474	2.506	1	.113	.004		

a variable(s) entered on step 1: age_range4, obesity_severity2, Ethnicity2, hist_gest_diab, fam_hist_diab, ges_age_deliv, apgar_score3, apgar_range2, weight_baby_ranges.
